# Interleukin 6 associates with reduced grey matter volume and resting-state connectivity in the anterior cingulate cortex in bipolar patients

**DOI:** 10.1016/j.bbih.2022.100522

**Published:** 2022-09-24

**Authors:** Benedetta Vai, Mariagrazia Palladini, Cristina Lorenzi, Raffaella Zanardi, Sara Poletti, Veronica Aggio, Francesco Benedetti

**Affiliations:** a Psychiatry and Clinical Psychobiology, Division of Neuroscience, IRCCS Ospedale San Raffaele, Milano, Italy; b University Vita-Salute San Raffaele, Milano, Italy; c Unit of Mood Disorders, IRCCS Ospedale San Raffaele- Turro, Milano, Italy

**Keywords:** Bipolar disorder, Depression, Interleukin 6, Grey matter, Anterior cingulate cortex, Resting state

## Abstract

High levels of peripheral IL-6, a pro-inflammatory cytokine, have been indicated as a key element of the bipolar disorder (BD), allowing to differentiate BD from major depression with high accuracy and to early detect poor responders to antidepressant treatments. IL-6 may contribute to BD pathophysiology through its effects on the neurobiological underpinnings of the disorder, such as grey matter (GM) volumes and resting state functional connectivity (rs-FC) abnormalities. In this study, we primary investigate the relationship between the peripheral plasmatic level of IL-6 and GM volumes, obtained with Voxel-Based Morphometry, in 84 BD inpatients. As secondary aims, we explored if IL-6 levels may be related to self-reported psychopathological dimensions of depression (i.e. symptoms severity and cognitive biases) and seed based rs-FC of brain regions structurally associated with the cytokine. Results showed that higher level of peripheral IL-6 was associated to lower GM volumes in supragenual anterior cingulate cortex, and reduced rs-FC between this area and medial orbito-frontal cortex in BD. Furthermore, in depressed patients IL-6 positively correlated to cognitive biases typically associated to depressive episodes, such as the perceived uncontrollability of negative events, or their generalization across future and situations. Our data provide additional evidence of detrimental effect of systemic inflammation on brain structure in BD and confirm the crucial role of anterior cingulate cortex as neural underpinning of the disorder. However, future studies are needed to replicate our findings in larger samples.

## Highlights

• In BD, IL-6 was correlated with lower grey matter in anterior cingulate cortex.
• IL-6 was associated with reduced rs-FC between ACC and orbito-frontal cortex.
• IL-6 was positively correlated with depressive cognitive biases.

## 1 Introduction

High levels of peripheral IL-6, a pro-inflammatory cytokine, have been indicated as a key element of the bipolar disorder (BD) (Lu et al., 2019a), allowing to differentiate BD from major depression with high accuracy (Poletti et al., 2020) and to early detect poor responders to antidepressant treatments (Benedetti et al., 2017).

IL-6 has been involved in the differentiation of specific T- and B-cell subsets, regulating the balance between Th17 cells and regulatory T cells by promoting Th17 differentiation and inhibiting Treg differentiation, thus, leading to an imbalance towards Th17 responses away from Treg, which is associated with inflammatory and autoimmune conditions (Kimura and Kishimoto, 2010). IL-6 mainly involved in inflammation and immune activation, inducing and maintaining inflammatory diseases, it also regulates the acute phase response, and the availability of tryptophan for serotonin synthesis (Kishimoto, 2005).

In healthy controls, peripheral levels of IL-6 were associated with decreased grey matter volume (Jefferson et al., 2007), reduced white matter integrity in corpus callosum (Bettcher et al., 2014), lower resting-state connectivity (rs-FC) (Marsland et al., 2017) and lower anterior cingulate cortex (ACC) FC during emotional processing (Harrison et al., 2009). In drug naïve MDD patients, serum level of IL-6 inversely correlated with bilateral superior frontal and medial orbitofrontal cortical thickness (Kakeda et al., 2018). In BD, higher levels of IL-6 soluble receptor (sIL-6R) were associated with lower cortical thickness in the middle temporal gyrus (Tu et al., 2017), lower grey matter volumes in cerebellum, striatal, parietal, temporal, and frontal cortexes (Bai et al., 2020), and abnormal rs-FC between medial prefrontal cortex and other regions deeply involved in mood regulations such as amygdala, hippocampus, insula, putamen, pallidum, and subgenual ACC (Tu et al., 2017). While IL-6 was also inversely related to rs-FC between the insula and postcentral cortex in depressed unmedicated BD patients (Chen et al., 2020). However, to the best of our knowledge, no previous studies explored if IL-6 peripheral level may be associated with reduced grey matter volumes in BD. This disorder has been extensively associated with abnormalities in corticolimbic grey matter volumes (Ganzola and Duchesne, 2017; Lu et al., 2019b), also paralleled by dysregulation of resting-state functional networks (Vai et al., 2019a).

In this study, we primary aimed to investigate the relationship between the peripheral level of IL-6 and GM volumes in 84 BD inpatients. As secondary aims, we explored if IL-6 levels may be related to self-reported psychopathological dimensions of depression (i.e. symptoms severity and cognitive biases) and rs-FC of brain regions structurally associated with the cytokine.

## 2 Materials and methods

We studied 84 inpatients affected by BD, type I, at Psychiatric Clinic “IRCCS San Raffaele Turro” of Milano, recruited over the last seven years until December 2018. BD diagnosis and clinical assessment were performed by trained psychiatrists according to the DSM-IV TR. Exclusion criteria were presence of major psychiatric co-diagnoses, mental retardation, history of drug or alcohol dependency or abuse within the last six months, major medical/neurological disorders and inflammatory disorders, and pregnancy. After a complete description of the study, written consent was obtained.

We assessed clinical features, including the number of previous episodes, the overall duration of illness in years and current mood phases (euthymia, mania, or depression), Body Mass Index (BMI), and actual and past pharmacotherapy.

Depressed patients’ current symptomatology was evaluated through the Beck Depression Inventory-13 (BDI) (Beck et al., 1996), while Cognitive biases related to depression through the Cognition Questionnaire (CQ) (Fennell and Campbell, 1984), a self-report measure that assesses five dysfunctional attitudes of negative thinking: emotional impact, attribution of causality, generalization across time, generalization across situations, and perceived uncontrollability.

Blood sampling for cytokine assessment was performed in the morning, and plasma was stocked between −60 and −80 °C and levels of analytes were measured within 32 months, using a bead-based Luminex system based on xMAP technology (Bio-Rad Laboratory, Hercules, CA, USA). Intra- and inter-assay coefficient of variation were calculated for standards and for a subsample assessed in all the plates, and ranged from 2.10 to 18.18% in agreement with reported observation for Luminex (Wolf et al., 1998). IL-6 quantification was extracted by the Bio-Plex Pro Human Cytokine 27-plex panel.

The concentration of each analyte was obtained by interpolating FI to a dilution standard curve over at least 7 dilution points supplied with the kit and calculated using a 5 PL curve by the Bio-Plex Manager software (Bio-Rad). When the observed plasma levels were below the sensitivity threshold of the method, they were replaced with imputation values (lower limit of quantification for each cytokine). Intra- and interassay coefficient of variation was calculated for standards and a subsample assessed in all the plates and ranged from 2.10 to 18.18% in agreement with reported observations.

All the patients underwent MRI scan. T1-weighted images were acquired on two 3.0 T scanners (the hospital scanner was updated in October 2016): i) until June of 2016 - we acquired 43 BP with Gyroscan Intera, Philips, Netherlands employing an 8 channels SENSE head coil (T1-weighted MPRAGE sequences: TR 25.00 ms, TE 4.6 ms, field of view FOV = 230 mm, matrix = 256 × 256, in-plane resolution 0.9 × 0.9 mm, yielding 220 transversal slices with a thickness of 0.8 mm), ii) from October 2016 to December 2018–41 BP acquisitions were obtained with the new scanner 3T Ingenia CX, Philips, The Netherlands using a 32-channel sensitivity encoding SENSE head coil (T1-weighted MPRAGE sequence: TR 8.00 ms, TE 3.7 ms, field of view FOV = 256 mm, matrix = 256 × 256, in-plane resolution 1 × 1 mm, yielding 182 transversal slices with a thickness of 1 mm). Images were processed by using Computational Anatomy Toolbox (CAT12) using default pipeline in Matlab R2016b. Notably, the tissue segments were normalized to a customized template, defined as an average template of the studied sample. The resulting template was then registered to MNI space. GM images were smoothed with an 8-mm full width at half maximum Gaussian filter. At second level, we investigated the effect of IL-6 on grey matter volumes, while controlling for nuisance covariates such as episode frequency (calculated as total number of episodes on overall duration), age, sex, BMI, phase of illness, scanner (0 vs 1), and Total Intracranial Volume as nuisance covariates. Analysis was performed whole brain and Family Wise Error (FWE) corrected for multiple comparisons at the cluster level (p_FWE_<0.05, initial uncorrected threshold p < 0.001). Absolute masking of 0.1 was applied. Furthermore, considering the potential effect of plausible outliers in IL-6 levels, we extracted individual grey matter volumes in significant clusters through Marsbar, and performed post-hoc robust regressions in R (RStudio, 2022.02.0, package robustbase, function lmrob), including all the previously considered variables in the model.

Functional MRI images obtained in resting-state condition were acquired in a subset of 15 participants (17.86% of the whole sample). Subjects were instructed to keep their eyes closed, and not to think about anything in particular – they simply had to let their mind wander – while do not to fall asleep. This sample was acquired with Ingenia CX, Philips, The Netherlands, with gradient-echo echo-planar images (EPI) 32-channel sensitivity encoding (SENSE) head coil. 200 T*2-weighted volumes were obtained [repetition time (TR) = 2000 ms, echo time (TE) = 30 ms, flip angle = 85°, field of view = 192 mm, number of slices = 38, slice thickness = 3.7 mm, matrix size = 64 × 62 reconstructed up to 96 × 96 pixels]. Overall, data acquisition lasted 6 min and 56 s. The information generated by imaging acquisition was then imported into CONN toolbox (www.nitrc.org/projects/conn) including default preprocessing pipeline. Scrubbing detected one subject with more than 20% of detected outliers volumes, he was removed from the subsequent analyses. A band-pass filter (0.01 Hz ∼ 0.1 Hz) and linear detrending were applied to remove linear drift artifacts and high-frequency noise. As first-level analyses, we performed seed-based analysis by computing the correlation between the time-course signal in a seed region with the rest of the voxels in the brain. In particular, our seed was defined by using second-level results from VBM analysis, previously binarized as a mask. A Fisher transformation and Weighted General Linear Model approach were applied. In second-level analyses, we explored the effect of IL-6 on seed-based connectivity. Analysis was performed whole brain and Family Wise Error (FWE) corrected for multiple comparisons at the cluster level (pFWE<0.05, initial uncorrected threshold p < 0.001). As for grey matter volumes, we extracted connectivity parameters, and performed a post-hoc robust regression to confirm the results.

In depressed patients, associations between IL-6 and self-reported psychopathology (BDI-13 and CQ) were investigated through Pearson correlations in StatSoft Statistica 8.0 (Tulsa, OK, USA). Significant threshold was p < 0.05, also corrected for multiple comparisons by using FDR procedure.

## 3 Results

The demographic and clinical data of 84 BD patients are reported in Table 1. Sixty-four patients were in depressive phase (75%), 16 in manic phase (18%), and 4 in euthymic phase (4.7%). For IL-6, only 3 subjects were imputed because below the detection level (3.57% of the sample). A total of 43 subjects were acquired with Gyroscan Intera, Philips 3T scanner (51.19%), while 41 with the Ingenia CX, Philips (48.81%).

**Table 1:** Descriptive statistics. Statistics are referred to comparison between depressed, manic, and euthymic patients. CQ, Cognitive questionnaire, Mean ± Standard Deviation, NA, Not available.

	Whole sample (n = 84)	Depressed patients (n = 64)	Manic patients (n = 16)	Euthymic patients (n = 4)	t, F or χ^2^	p- value
**Age**	47.01 ± 11.85	46.39 ± 11.52	52.31 ± 11.41	37.25 ± 12.61	3.21	0.04567
**Sex -** Male (Female)	29 (55)	17 (47)	10 (6)	2 (2)	7.76	0.02067
**Interleukin 6**	22.38 ± 48.69	20.98 ± 48.4	24.23 ± 46.69	37.52 ± 71.752	0.23	0.79730
**Frequency of mood episodes**	0.83 ± 0.87	0.85 ± 0.89	0.86 ± 0.91	0.46 ± 0.37	0.36	0.69716
**Duration of illness**	17.52 ± 11.15	17.69 ± 10.86	17.50 ± 12.69	15.00 ± 12.11	0.11	0.89861
**Number of mood episodes**	9.00 ± 8.11	9.36 ± 8.06	8.63 ± 9.18	4.75 ± 2.63	0.62	0.53893
**Total intracranial volume**	1375.12 ± 216.65	1378.90 ± 165.76	1400.32 ± 145.55	1532.31 ± 231.31	1.66	0.19644
**Beck Depressive Inventory – Total score**	NA	12.78 ± 8.71	NA	NA	–	–
**CQ1** **Emotional Impact**	NA	3.67 ± 2.95	NA	NA	–	–
**CQ2 Attribution Of Causality**	NA	5.43 ± 3.07	NA	NA	–	–
**CQ3** **Generalization Across Time**	NA	4.05 ± 2.95	NA	NA	–	–
**CQ4** **Generalization Across Situations**	NA	5.90 ± 2.77	NA	NA	–	–
**CQ5** **Perceived Uncontrollability**	NA	3.67 ± 2.80	NA	NA	–	–
**CQ - Total score**	NA	22.71 ± 11.62	NA	NA	–	–

**Fig. 1:**
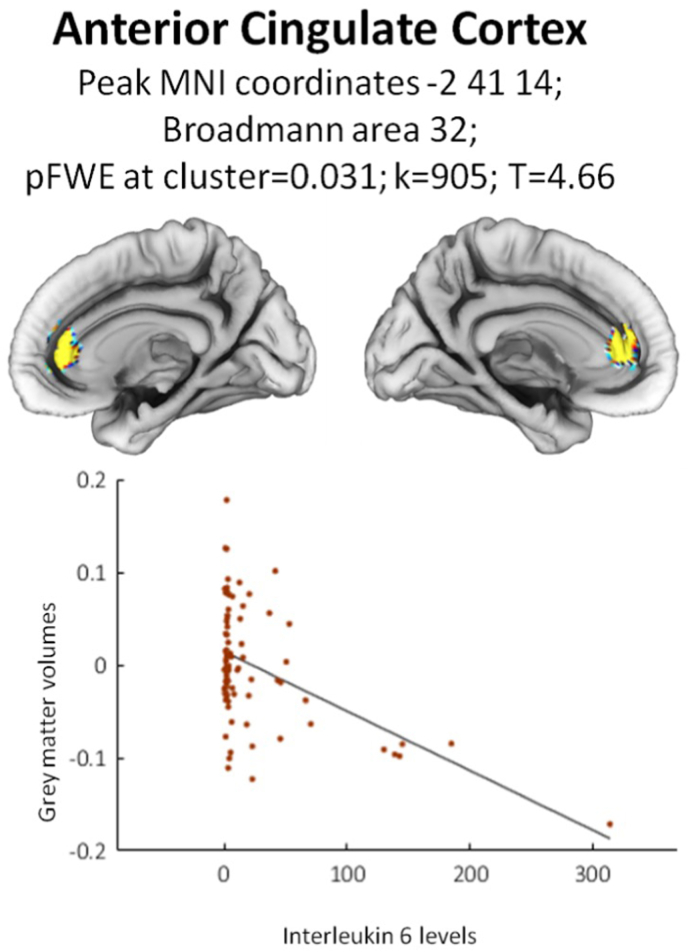
Effect of IL-6 on grey matter volumes in Anterior Cingulate Cortex.

As a result of structural VBM analyses, IL-6 negatively affected grey matter volumes in Anterior Cingulate Cortex (Broadmann area 32, MNI coordinates −2 +41 + 14, k = 905, p_FWE_ = 0.031; Fig. 1) in the whole sample. Robust regression confirmed a significant effect of IL-6 on ACC grey matter volume (coef = −0.0005, Std. Err = 0.00006, t = −7.8, p < 0.0001). Therefore, this cluster was extracted to define a specific ACC mask and entered as a seed region for the following Resting-State connectivity analysis.

Functional connectivity analyses in 14 subjects (4 Males, Age Mean ± Standard Deviation, 43.21 ± 15.31, 12 depressed, 2 manic, IL-6 13.22 ± 18.61) showed IL-6 to decrease resting-state functional connectivity between ACC and medial orbitofrontal cortex (OFC, Broadman area 11, MNI coordinates +8 + 34 -22, k = 146, p_FWE_ = 0.012) in the whole sample. Robust regression confirmed a significant effect of IL-6 (coef = −0.008, Std. Err = 0.0007, t = −12.1, p < 0.0001).

In depressed patients (n = 34), IL-6 was positively related to depressive cognitive distortions: (i.e. generalization across time (r = 0.44, p = 0.009, q = 0.029), generalization across situations (r = 0.43, p = 0.01, q = 0.029), perceived uncontrollability (r = 0.51, p = 0.002, q = 0.016).

## 4 Conclusion

We showed that a higher level of peripheral IL-6 was associated to lower GM volumes in supragenual ACC, and reduced rs-FC between this area and medial OFC in BD. ACC exert a crucial role in the pathophysiology of mood disorders: its GM volume reduction is one of the most consistent neural underpinning detected in BD (Lu et al., 2019c), and, thanks to its connections within the corticolimbic network, it is deeply involved in either voluntary or automatic regulation of emotional response (Vai et al., 2019b). Furthermore, in our sample of depressed patients IL-6 positively correlated to cognitive biases typically associated to depressive episodes, such as the perceived uncontrollability of negative events, or their generalization across future and situations.

In line with our results, previous studies associated IL-6 to decreased total brain volume (Jefferson et al., 2007; Marsland et al., 2008), and cortical atrophy in healthy controls (Baune et al., 2009), as well as in MDD drug naïve patients with reduced thickness in prefrontal cortex, a brain region characterized by a high expression of IL-6 receptors (Vitkovic et al., 2000), (Kakeda et al., 2018), in particular in ACC (Jefferson et al., 2007; Kakeda et al., 2018). These negative associations, as we detected, may be explain by e a receptor-mediated neurotoxic effect of IL-6 on GM volumes (Kakeda et al., 2018). In BD, previous findings also confirmed that sIL-6R levels were related to GM reduction in frontal cortex (Bai et al., 2020). Furthermore, transient or persistent alterations of blood-brain barrier ‘s permeability may facilitate access of cytokines from periphery to the central nervous system, thus triggering microglia activation (Patel and Frey, 2015). Under a chronic inflammatory state, as suggested in BD, microglia may, in turn, solicit release of several cytokines in loco, such as IL-6, TNF-α and IL-1β, can be responsible for glial cell loss and activation of apoptotic cascade (Monje et al., 2003), which may contribute to reduction in cortical volume. Potential detrimental effects of IL-6 on brain morphometry are also corroborated in animal models where increased levels of proinflammatory cytokines, such as IL-6, were associated with neurodegeneration (Campbell et al., 1993), and detrimental effects on long-term potentiation (Bellinger et al., 1995). IL-6 also influences several physiological processes involved in BD, that may also indirectly affect prefrontal cortical volumes and functioning, including the regulation of hypothalamic-pituitary-adrenal axis, noradrenergic, serotoninergic and glutamatergic transmission, kinase signaling, and oxidative stress (Müller and Schwarz, 2007; Meng et al., 2020; Mastorakos et al., 1994; Anderson et al., 2014). All these processes, related a suggested chronic inflammatory state in BD and high levels of IL-6, can contribute to reduction in ACC volume detected in our sample.

Our findings also showed a negative association between higher IL-6 and lower functional connectivity between ACC and OFC, in line with previous results in healthy controls, where increases of plasmatic IL-6 were associated with a reduction of functional connectivity between the medial prefrontal cortex and subgenual ACC during emotional processing (Harrison et al., 2009). Abnormal rs-FC between these regions, strongly connected among themselves also in resting-state condition as part of the ‘default mode network’, have been previously found in BD across mood states (Vai et al., 2019b).

Finally, we detected an association between IL-6 and negative attributional cognitive bias of depression. Associations between inflammation and negative cognitive biases were previously showed in somatic illnesses (Cooper et al., 2018), and mood disorders (Niemegeers et al., 2019). Concerning IL-6, baseline peripheral levels of this cytokine also allowed predicting cognitive symptoms of depression at 12 years follow-up, suggesting that IL-6 can precede depression, at least with regard to its cognitive symptoms (Gimeno et al., 2009). IL-6 was also associated with worse cognitive performance even in absence of a diseases (Singh-Manoux et al., 2014). Likewise, attentional cognitive biases result weakened in patients who received antidepressant therapy (Godlewska et al., 2016), which in turn acts as regulator of inflammatory state (Kalkman and Feuerbach, 2016). Altogether, available evidence points towards immuno-inflammatory pathways fostering cognitive distortions in affective disorders. According to the paradigm of cytokine-induced sickness behaviour in depression phenomenology (Dantzer, 2009), it can be hypothesized the effect of that pro-inflammatory mediators in modulating subgenual cingulate activity and meso-limbic connectivity contributes to bias cognition, as detectable in depression. Further supporting this notion, updated cognitive models of depression posit a major implication of ACC in mood-congruent cognitive bias, mainly related to its crucial role in attentional control and coordination in emotional processing (Noworyta et al., 2021).

However, relatively small sample size may raise issues related to power, generalizability, and possible population stratification. Future studies should replicate our findings, also exploring possible associations with other cytokines related to immunoinflammatory processes in BD.

Despite limitations, our data showed that IL-6 was inversely related to GM volume and rs-FC in ACC paralleled by higher cognitive depressive biases, providing additional evidence of a detrimental effect of systemic inflammation on brain structure in BD and confirming the crucial role of ACC in the neurobiology of the disorder.

## Role of the funding source

This work was supported by the 10.13039/501100003196Italian Ministry of Health, grant RF-2011-02350980; and by H2020-EU.3.1.1 grant 754740 MOODSTRATIFICATION.

## Declaration of competing interest

None.

## Data Availability

The data will be available upon request and with the approval of our ethics office.
